# Did Mendel breed mice?

**DOI:** 10.1007/s40656-026-00760-3

**Published:** 2026-09-11

**Authors:** Péter Poczai

**Affiliations:** Finnish Museum of Natural History, and Department of Organismal and Evolutionary Biology, Faculty of Biological and Environmental Sciences, University of Helsinki, PO Box 7, 00014 Helsinki, Finland

**Keywords:** Heredity research, History of genetics, Matthew effect, Scientific hagiography, Whig history

## Abstract

Scientific legends are rarely born fully formed, and the story of Mendel’s mice is no exception. When the mice appear in the historical record, they do so through contradicting accounts on the most basic questions of what the animals looked like and what, if anything, was done with them. This story also reminds us that Mendel was not the isolated genius of popular imagination but a friar navigating a hostile bishop, considerable institutional pressures, and the deep suspicion with which the Church regarded the study of heredity in nineteenth-century Moravia. Whether or not he crossed those mice, the persistence of the story illustrates something broader about how scientific reputations are constructed and maintained. The various accounts converge on the same outcome, in which details that feel appropriate to a legend accumulate around it regardless of whether the evidence supports them. The mice of Mendel, plausible yet unverifiable and irresistible, are a particularly instructive example of these processes at work.

## Introduction

Few figures in the history of science have accumulated as many myths and anecdotes as Gregor Johann Mendel (1822–1884), and it is difficult to know quite where to begin when attempting to disentangle them. We tend to picture him in his cassock, bent over his experiments or moving quietly among his plants, and we are naturally drawn to the more heroic model of scientific discovery (Shortland & Yeo, 2009). Our relationship with science, and with the great intellectuals who shaped it, is often constructed in this way. The more prosaic details of their daily existence receive considerably less attention, despite frequently offering greater insight than any grand narrative (Sapp, 1990). It has been said that Mendel was not really such a magician (Kampourakis, 2024), and that the episodes recounted here, drawn from personal experience and passing conversation, may seem of little consequence beside his fundamental discoveries. Yet the smallest details sometimes reveal the most. Was it not the legendary apple, after all, that set Newton thinking about gravity (Fara, 1999)? And was it not a falling pea that first prompted Mendel to turn his attention to heredity? With that spirit in mind, one might reasonably ask whether Newton the mystic concerned himself with alchemy, or whether Mendel perhaps kept mice. It is precisely through such apparently mundane questions that we sometimes come closest to understanding who these figures actually were.

The mouse question arose, as such questions often do, at a conference, accompanied by the usual cluster of anecdotes about the monk in his garden (Henig, 2000) and data that seemed rather too clean to be entirely trusted (Franklin et al., 2008). The topic first reached me as a young researcher, possibly during a second-year genetics course, though at that stage I had not yet devoted serious thought to Mendel or to the history of genetics more broadly. It resurfaced some years later during a conversation about potato crosses with a professor, who had himself received the story from his own professor, who had in turn received it from somewhere else entirely. It was around this point that I began to suspect the mice had drifted into the territory of scientific urban legend. Certain small details continued to nag at me, however, and after several years the story drew me back in again (Mukherjee, 2016; Paigen, 2003; Phifer-Rixey & Nachman, 2015; Reese, 2009; van der Weyden et al., 2011).

## A famous man and his alleged mice

The mice of Mendel proved to be the most stubborn of all the puzzles encountered in this investigation, and what follows is an attempt to lay out the evidence clearly enough to spare any future researcher the same circuitous journey. The earliest and, it must be said, the only source for the story of Mendel and his mice is Hugo Iltis (1882–1952), a successor to Mendel as a teacher in Brno, who returns to the subject twice in his *Life of Mendel*.^1^ By his account Mendel’s lodging in the abbey consisted of two rooms, one serving as his living quarters, the other given over to a small private menagerie, an unspecified collection of birds and, more tellingly, a „proper mouse breeding operation" (Iltis, 1924, p. 57). Two of Mendel’s contemporaries vouched for the arrangement, his former pupil the accountant Franz Hornisch and his *Oberrealschule* colleague Inspector Nowotny^2^ (Iltis, 1924, p. 57). What filled the young Hornisch with wonder was not that the mice were white but that ordinary grey ones were kept among them as well, and Nowotny for his part confirmed the fact.

This is a useful moment to reflect on what the Iltis biography actually represents. It remains the only account constructed from direct, in-person interviews with individuals who had known Mendel personally (Dunn, 1965a). Iltis sought out those still living who could speak from memory, and he recorded what they told him. That is both the considerable strength of the book and its fundamental limitation. Much of the evidence is unverifiable precisely because it was never written down at the time; it passed through conversation and memory before reaching the page. It is against this uncertain background that Iltis ventures a further suggestion, and he is careful to flag it as his own speculation, that Mendel may have crossed the mice informally, as much out of curiosity as scientific intent (Iltis, 1924, p. 68). He acknowledges that such activity would not necessarily have carried any rigorous experimental weight. Yet he cannot quite resist adding that a man of the disposition of Mendel, having set crosses in motion, would almost certainly have noticed the behavior of dominant and recessive traits in the offspring. This, after all, was Mendel. The more pressing question is why Mendel himself left no record of any of it. Iltis reads the silence as deliberate caution. In certain ecclesiastical quarters, a priest who pursued the natural sciences was already regarded with a degree of suspicion. A priest who conducted breeding experiments with animals risked being seen as transgressing something more fundamental. Mendel had reasons to be careful, and those reasons had a face: the bishop was not well disposed toward him.

## A diocese in difficulties

It is worth pausing here to take stock of what can and cannot reasonably be concluded. That Mendel kept mice is plausible enough, and there is no compelling reason to doubt it, what Mendel actually did with them remains genuinely open. For all the surviving evidence tells us, they may have served no experimental purpose whatsoever. In the absence of any direct proof, Leslie Dunn (1965b) remained appropriately skeptical, and Alfred Sturtevant (1965) was no more persuaded by the supposed mouse experiments than the documentary record warrants.

What is not in doubt is the environment in which all of this unfolded. The Bishop of Brno, Anton Ernst von Schaffgotsch (1804–1870), harbored a settled antipathy toward Mendel and toward St. Thomas’s Abbey as a whole. He had recommended in his report, forwarded to Rome by Cardinal Schwarzenberg, that the Holy See dissolve the establishment entirely, having arrived at the firm conviction that the abbey had been claimed by what he called the enemy of mankind (Huber, 1978).^3^ Bishop Schaffgotsch’s report should be read for what it was, a hostile brief assembled to place the monastery in the worst possible light and to furnish grounds for its suppression. Its charges are best reported as his allegations rather than as established fact. One of the friars, Aurelius Thaler, was said to have delivered blasphemous remarks to his students while intoxicated, an accusation the more remarkable because Thaler had died more than a decade before the report was drawn up (Fairbanks, 2022, p. 44). Another, Anselm Rambousek, was alleged to have been seen bathing nearly naked in public. Philipp Nedéle had indeed been removed from his chair of biblical studies by imperial decree for rationalist exegesis, and Matthäus Klácel, the most radical intellect of the house, had taken part in the Slav Congress of 1848 and edited newspapers that drew repeated episcopal censure. Thomas Bratránek was described as long inwardly estranged from the Church, while Anton Alt and Philipp Gabriel were faulted for pursuing wholly secular academic careers. Even the celebrated musical work of Paulus Křížkovský was pressed into service as evidence of a community sunk in worldly life. The bishop was not a man inclined to generosity of interpretation, and he marshalled these episodes with evident purpose. Yet the report will not bear the weight its author wished to place on it. Its bias is plain, and the document reads as a malicious and misleading construction rather than a sober account (Fairbanks, 2022, p. 46). In the event the bishop did not have his way, and the monastery was not closed.

On the broader question of ecclesiastical attitudes toward heredity research, Iltis is on firmer ground. The study of inheritance had typically proceeded through inbreeding, and inbreeding combined with deliberate selection had an uncomfortable proximity to questions the Church preferred to leave undisturbed. Matters reached a particular sensitivity in Brno when the secretary of the local scientific society, Christian Carl André (1763–1831), compared the selective breeding of sheep to the perpetuation of the noble Habsburg jaw and nose (André, 1819), and compounded the offense by publishing a caricature of the comparison in his *National Calendar* (André, 1821; Poczai et al., 2022). The Church responded by tightening its oversight of the intellectual activities of the city, and instruction in the propagation of organic bodies by means of two sexes, on the characteristics and selection, was sharply reduced.^4^ As for Mendel personally, the objection of the bishop was direct and institutional. Mendel, in his view, had studied profane sciences at a worldly establishment in Vienna, at the expense of the abbey, for the purpose of becoming a professor of those same sciences at a state institution (Huber, 1978). That alone was sufficient grounds for disapproval.

## The chuckle that wasn’t

By the turn of the millennium, the mice had settled into a comfortable position in the historiography of genetics. The claim had taken hold that Mendel began with mice, observed segregation ratios in fur color, and only switched to peas when a disapproving bishop decided it was improper for a friar to watch rodent copulation. Daniel Fairbanks (2022) traced the crystallization of this narrative to *The Monk in the Garden* by Robin Marantz Henig (2000), which elaborated considerably on Iltis, added a bargain between the abbot, Cyrill Franz Napp, and Bishop Schaffgotsch, and appended a remark attributed to Mendel, delivered with a chuckle, that he had turned to plants because the bishop did not understand that plants also have sex. There is no evidence Mendel ever said anything of the kind. It is a literary invention that entered scientific circulation and was subsequently repeated in journals and books of considerable standing.

Several problems attend the wider story. Mendel was not a monk but an Augustinian friar, a distinction that quietly shapes how the narrative is framed, and one that has caught out more than one historian, including the present author once. The man who wrote from Vienna that he would rather be in the primeval forest than attending pious exercises, and who spent years in stubborn institutional combat over a tax on religious properties, was not obviously someone who would abandon promising experiments to avoid episcopal suspicion (Weir, 1968). The lands of the abbey supported animal husbandry alongside horticulture, so the observation of animal reproduction was hardly a novelty. Weir (1968) offered the most plausible alternative explanation: anyone who has maintained mice in any number will confirm that it is the animals themselves that offend, and the friends of Mendel rather than his enemy the bishop may have hastened the end of the experiments. Everything else remains speculation.

## On the dangers of a perfect prelude

**Fig. 1:**
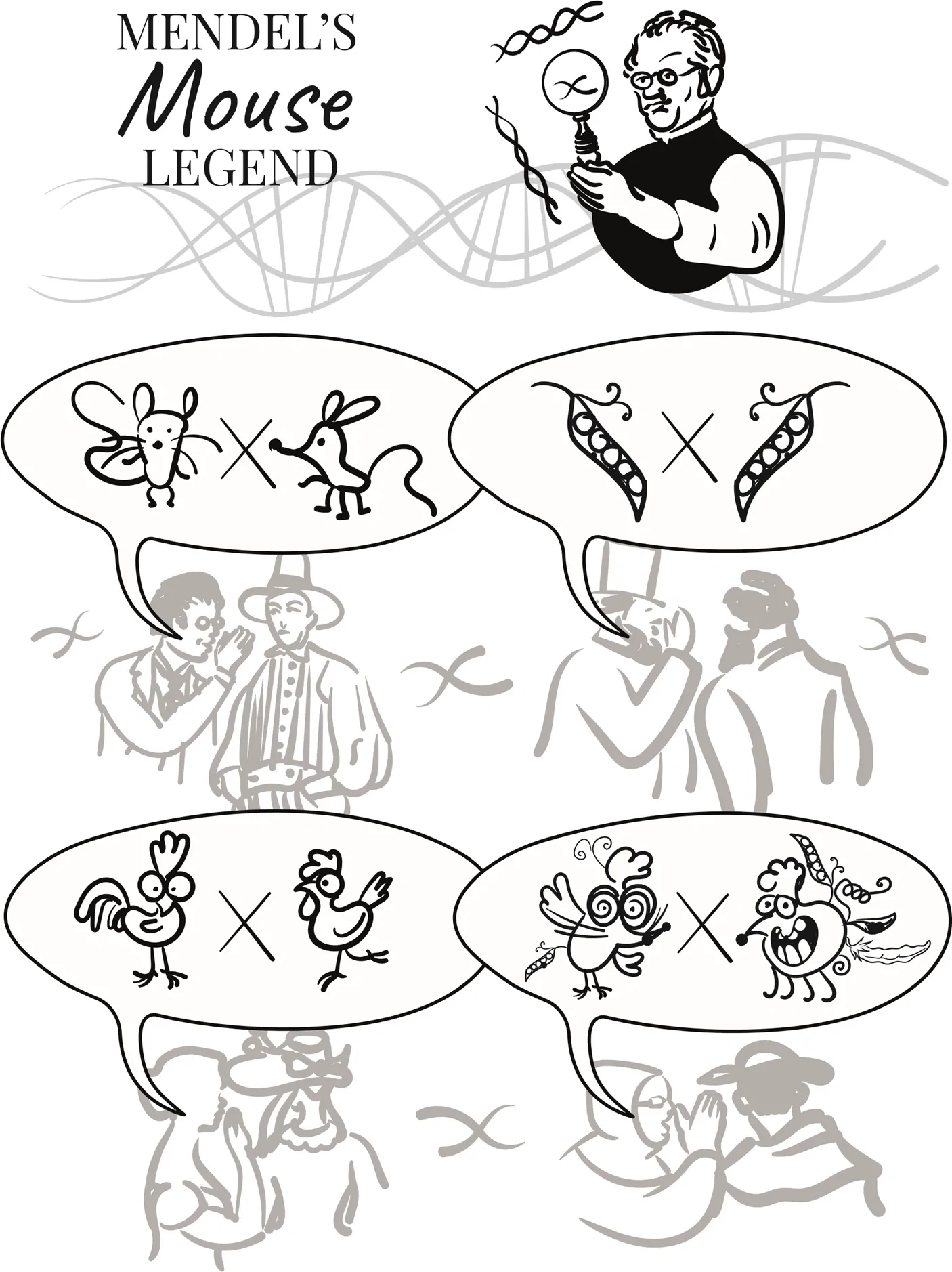
Mendel's mouse legend, a tongue-in-cheek take on how the story grows in the retelling. Iltis reported that Mendel kept mice and may have crossed them (top left), yet the experiments he actually published were on peas (top right). Playfully extending the legend to caged birds beside the mice might (bottom left), while the persistent but unfounded tale has the Church's unease at experiments on animal reproduction bringing the crossings to an end (bottom right). The faint figures behind the panels are the pupils, colleagues and professors through whom such anecdotes pass until their origins vanish, the Matthew effect at work

Like the apple of Newton, the story of the mice of Mendel is perhaps best understood as an instance of what might be called Mendelian hagiography, the process by which later generations render discovery narratively intelligible by populating a life with details that feel appropriate to the legend. The mice, whether real, imagined, or quietly embellished in the retelling, function to stabilize the scientific reputation of an already canonized figure, accumulating around his biography with the same gravitational logic that draws anecdotes toward famous names regardless of whether the evidence supports them (Golinski, 2005). There is no particular reason why Mendel, a friar of a real abbey living a real and considerably complicated life, should be exempt from what Robert K. Merton identified as the Matthew effect (Fig. 1), the well-documented tendency of scientific communities to concentrate credit, stories, and legendary association around figures already celebrated, passing anecdotes from professor to professor until their origins become impossible to trace (Merton, 1968). We read the past through the lens of what we already know, selecting and amplifying those details that appear to anticipate later triumphs while quietly setting aside those that do not, which is precisely the Whig history pattern at work in the mouse story (Butterfield, 1931).

It must be admitted that the temptation is considerable. There is something almost irresistible about the image of Mendel observing segregation ratios in those small fur-coated creatures that would later become among the most widely used laboratory organisms and standard subjects of genetic investigation. They would make a perfect prelude, a foreshadowing in miniature of everything the pea experiments would confirm. The thought is seductive enough that it has led more than one historian down that path. And once one starts looking at it that way, it becomes difficult not to notice that the birds in the cage beside the mice in the room of Mendel might, for all we know, have been chickens, which would later become the organism of choice of William Bateson. One could construct quite a pleasing origin story from that. Which is, of course, precisely the problem.

## Data Availability

This article is a historical and historiographical analysis and generated no new data or software code. All sources consulted are cited in the Literature Cited. Archival material referenced is held at the Moravian Archive (Moravský zemský archiv) in Brno, Czech Republic, under the signatures given in the text.

## Footnotes

1 The anecdote appears in fuller form in the German original, where Iltis describes both the birds and the mouse colony and, at the second passage, adds his own conjecture on its bearing for the genesis of the experiments (Iltis 1924, pp. 57, 68). The English translation is neither as complete nor internally consistent on this point. At the corresponding place it records only ordinary mice in Mendel's rooms (Iltis 1932, p. 92), yet a later passage reverts to the fuller version and speaks of both white and grey mice (Iltis 1932, p. 105), thereby contradicting the earlier page. A reader relying on the 1932 translation alone will therefore meet a thinner and partly self-contradictory account, and the German text of 1924 should be treated as the primary witness.

2 Iltis refers to this colleague only as Inspektor Nowotny, without a first name (Iltis 1924, p. 57). Klein and Klein (2013, p. 381, note 161) give the name as Adolph but cite no source, and Fairbanks (2022, p. 192, note 7) could find no Adolph Nowotny (or Nowotný) in the membership rolls of the *Verhandlungen des naturforschenden Vereines in Brünn*, which for the years of Mendel's membership list only Johann Nowotný and Carl Nowotny. The first name therefore remains unverified.

3 The visitation report survives in the Archivio Apostolico Vaticano [previously Archivio Segreto Vaticano], *Sacra Congregatio Episcoporum et Regularium, Visitatio apostolica per imperium austriacum extra Italiam* 354 and 355, *Visita a Austria, Polonia* 1851 to 1859, *Varie* 4. Orel (1975) first reported the visitation, and Huber (1978) subsequently published a transcription of the text. The report was written in Latin, of which Czihak and Sladek (1991) later published a partial German translation, while Klein and Klein (2013) surveyed the visitation more broadly, as well as Fairbanks (2022) in *Chapter* 4.

4 Moravian Archive (MZA) in Brno, Czechia, Archivestand 13, Sign. P113/8.
